# Quantitative Proteome Analysis Reveals Changes in the Protein Landscape During Grape Berry Development With a Focus on Vacuolar Transport Proteins

**DOI:** 10.3389/fpls.2019.00641

**Published:** 2019-05-15

**Authors:** Liuqing Kuang, Shangwu Chen, Yan Guo, Huiqin Ma

**Affiliations:** ^1^Department of Fruit Tree Sciences, College of Horticulture, China Agricultural University, Beijing, China; ^2^College of Food Science and Nutritional Engineering, China Agricultural University, Beijing, China; ^3^College of Biological Sciences, China Agricultural University, Beijing, China

**Keywords:** comparative quantitative proteome, differentially abundant protein, grape development and ripening, intact vacuole isolation, transport protein, vacuole proteome, *Vitis vinifera* L.

## Abstract

The vacuole plays a central role in fruit growth and quality formation, yet its proteomic landscape is largely unknown. In the present study, a protocol for isolating intact vacuoles from grape flesh tissue was successfully established. Quantitative proteome analysis identified 2533 proteins from five sampling dates along Cabernet Sauvignon berry development from stage I to III; among them, 1443 proteins were identified on all five sampling dates in at least two biological replicates per sample and were designated core proteome, and 1820 were recruited as differentially abundant proteins (DAPs) by sequential pairwise comparisons using arbitrary fold change of >1.5 and *P* < 0.05. Metabolism consistently constituted the largest category of identified proteins for both core proteome and DAPs, together with a consistently high proportion of protein-fate category proteins, indicating that the classic lytic functions of vegetative cell vacuoles are maintained throughout berry development; accumulation of metabolites involved in high sugar and other berry qualities in the late developmental stage added to the conventional lytic role of the flesh cell vacuoles. Overall increases in abundance of the DAPs were seen in the transporter proteins, membrane fusion/vesicle trafficking, and protein-fate categories, and decreased abundance was seen for DAPs in the stress, energy and cytoskeleton categories as berry development progressed. A very pronounced proteomic change was revealed between late stage I and mid stage II, with 915 increased and 114 decreased DAPs, demonstrating a significant surge of the vacuolar proteome underlying the rather static phenotypical and physiological phase. We identified 161 transport proteins with differential abundance, including proton pumps, aquaporins, sugar transporters, ATP-binding cassette transporters and ion transport proteins, together with organic compound transport proteins, the highest number and variety of berry tonoplast transporters found in grape proteome efforts to date. We further found a pre-positive increment of 96 transport proteins from the middle of stage II, before the berry undergoes its dramatic physiological changes at and following véraison. Our results are the first to describe the proteome of a vacuole-enriched preparation, toward understanding the functions of the largest compartment in berry cells during grape growth and ripening.

## Introduction

The grapevine (*Vitis vinifera* L.) is a major cash crop worldwide. Grape berry development can be divided into three major stages displaying a double sigmoid curve. The first rapid growth stage (stage I), lasting 30–35 days, is characterized by berry set and enlargement due to active cell division and expansion; the vacuole shows a large amount of water influx and notable accumulation of tartaric and malic acids (Conde et al., 2007). Stage II is a lag phase of 25–40 days, depending on the cultivar, with little or no berry size enlargement. Berries at this stage are green, hard and acidic (Coombe, 1992). Véraison marks the onset of berry ripening and the initiation of stage III, characterized by berry enlargement, significant color change for red cultivars, texture softening, aroma formation, pronounced sugar accumulation, and acid catabolism (Conde et al., 2007; Deluc et al., 2007). The final yield and quality of berries are formed during the 30–40 days of stage III, when most of the grape industry’s valued metabolites are stored in the berry cell vacuole.

There are two major types of vacuole in plants: lytic and protein storage (Carter et al., 2004). Lytic vacuoles are in vegetative tissues, whereas protein storage vacuoles are present in the seeds of leguminous plants and cereals (protein bodies). Several other types of vacuoles have also been reported: motor cells of *Mimosa pudica* pulvini contain tannin-rich vacuoles (Fleurat-Lessard et al., 1997); *Mesembryanthemum crystallinum* contains neutral vacuoles, regarded as protein storage vacuoles, that accumulate NaCl, together with acidic vacuoles sequestering malic acid in the mesophyll cells (Epimashko et al., 2004). In most cases, the plant cell has only one type of vacuole, but during specific developmental transitions, two types of vacuoles may coexist (Frigerio et al., 2008).

The vacuole is the largest organelle in the plant cell, potentially occupying up to 90% of the cell volume, especially in ripening fruit. Fruit growth and cell expansion have been found to rely more on vacuole enlargement and cytosolic space increment than on cell division (Shiratake and Martinoia, 2007; Hedrich et al., 2015). Moreover, the vacuole functions as a reservoir for short- and long-term storage of metabolites, signaling compounds and potentially toxic compounds (Martinoia et al., 2012). As for berries, the predominant function of flesh vacuole at ripening is as a reservoir for nutritional substances that will attract seed dispersers. The composition and concentration of substances stored in vacuoles vary dynamically with berry development; each of these substances is thought to be taken up by, or remobilized out of the vacuole by distinct transport proteins (Martinoia et al., 2007). In the process of berry ripening, the fruit flesh cell vacuoles accumulate high concentrations of sugars, organic acids and secondary metabolites, distinguishing them from vegetative cell vacuoles.

Proteomic analysis is a powerful tool for identifying known and novel proteins on a large scale; proteomic studies have been carried out to explore developmental changes and explain the effects of abiotic and biotic stresses or of plant hormones on grape berry composition and product characteristics (Grimplet et al., 2009; Di Carli et al., 2011; Wang et al., 2012; Cramer et al., 2013; George et al., 2015). Specific tissues of physiological or commercial importance to berry quality, i.e., seed and skin, have also been isolated and subjected to proteomic studies (Deytieux et al., 2007; Negri et al., 2008; Martínez-Esteso et al., 2011). All of those studies addressed the whole-cell level and involved soluble proteomes; although of wide interest, few vacuolar proteins were identified, especially transport proteins. In fact, proteomic analysis of grape berry at the subcellular compartment level has never been reported, except for our earlier work on the plasma membrane of pre-véraison, véraison and stage III Cabernet Sauvignon berries, where 62 putative plasma membrane proteins were identified (Zhang et al., 2008). The tonoplast and vacuole proteomes of grape berries are largely unknown due to the obvious technical barriers to obtaining sufficient amounts of highly purified vacuoles; this holds true even for vegetative tissues of model plants with their physiologically stable, higher cell content (Fontes et al., 2010a,b).

To date, vacuole or tonoplast proteome studies have been mainly performed on the vegetative tissues of model plants. Intact vacuoles have been derived from *Arabidopsis thaliana* suspension and mesophyll cells, and the tonoplast or soluble-fraction proteins identified, revealing the proteins underlying the vacuolar function of lysis: glycosidase, stress-response proteins, and transporters of organic and inorganic molecules, proving that lysis is the major function of the large central vacuoles of these cells (Carter et al., 2004; Jaquinod et al., 2007). *Arabidopsis* mesophyll cell vacuoles were also isolated to characterize the function of tonoplast sugar transporters (TSTs) in sugar compartmentalization (Schulze et al., 2012). Vacuole isolation and protein identification have been carried out with barley mesophyll cells (Endler et al., 2006), young cells of cauliflower buds (Schmidt et al., 2007) and sugar beet taproot cells (Jung et al., 2015).

In grape, protoplasts and intact vacuoles have been isolated from berry suspension cultured cells (Fontes et al., 2010a,b); yet, aside from the valuable confirmation of vacuole integrity in the late stage of berry development (Fontes et al., 2010b), information on tonoplast and vacuolar protein populations is very limited, as no further detailed proteomic studies have been reported.

Here, we modified the protocol previously applied to grape berry suspension cultured cells to successfully isolate intact vacuoles from protoplasts derived from Cabernet Sauvignon berry mesocarp tissue on five sampling dates, covering all three berry developmental stages. A label-free quantitative proteomic approach revealed a significant vacuolar protein surge during the well-recognized lag stage of berry development. The highest number and diversity of tonoplast transport proteins to date were identified, and a high proportion of pumps, aquaporins, sugar transporters, and potassium transport proteins increased or maintained their high abundance from the middle of berry developmental stage II. Our results are the first to reveal dynamic changes in the vacuolar proteome during berry development and provide new insight into our knowledge of berry cell vacuolar function.

## Materials and Methods

### Plant Material

Berries of *V. vinifera* cv. Cabernet Sauvignon were collected from the Sino-French Demonstration Farm in Huailai (40°3′ N, 115°8′ W), China, in 2017. The soil type in the vineyard is sandy loam, vineyard management followed local practices and the vines were 16 years old when the samples were taken. At least 30 clusters were collected 30 days after flowering (DAF, late stage I), 52 DAF (middle of stage II), 66 DAF (late stage II, before véraison), 77 DAF (beginning of véraison), and 96 DAF (stage III), respectively, for berry physiological parameter assay and mesocarp cell vacuole isolation.

### Physiological Parameter Assay

Changes in transverse and vertical diameters during berry development were measured by digital vernier caliper. Soluble solids content was determined with a handheld refractometer (PAL-1, Atago, Tokyo, Japan). For each biological parameter, at least 50 berries were analyzed from different clusters every 7 days.

### Vacuole Isolation

Grape berry vacuoles were isolated by osmotic lysis of protoplasts following previously reported protocols (Fontes et al., 2010a,b) with some modifications. Three biological replicates were carried out for each of the five sampling dates. In brief, berries picked 30, 52, 66, 77, and 96 DAF were deseeded and peeled. The flesh was cut into cubes of approximately 3 mm^3^ and washed gently with washing buffer (255 mM KCl, 65 mM CaCl_2_, 1 mM DTT, 10 mM MES, pH 5.8) at 4°C, until the solution was clear; the final berry tissue cube washing buffer was kept at pH 5.8. Then 20 g of the flesh cubes was incubated for 45 min at 25°C under very gentle agitation (15–25 rpm) with washing buffer containing 1.5% (w/v) cellulase R-10 (Yakult Honsha, Tokyo, Japan), and 0.3% (w/v) pectolyase Y-23 (Yakult Honsha). The resulting protoplasts were gently collected, filtered through 0.3-mm pore size mesh and centrifuged for 5 min at 30 *g*. The protoplast pellet was washed twice with 4°C-precooled washing buffer, then lysed in a 2.5 volume of lysis buffer prewarmed to 42°C and containing 0.2 M mannitol, 10% (w/v) Ficoll, 15 mM EDTA, 2 mM DTT, 10 mM HEPES, pH 8.0; the mixture was gently stirred for 2 min and incubated for 10 min to release intact vacuoles from the protoplasts.

Vacuole purification was performed by three-step Ficoll-gradient centrifugation. The discontinuous gradient was prepared as follows: a bottom layer of the lysis mixture (10% Ficoll), middle layer of 4.0% Ficoll and top layer of vacuole buffer containing 0.5 M mannitol, 10 mM HEPES, pH 7.5, in the proportion of 5:3:1 (v/v). The 4.0% Ficoll solution was lysis buffer diluted 2:3 (v/v) with vacuole buffer. The 4.0% Ficoll solution and the vacuole buffer were gently laid on top of the protoplast lysate in the lysis buffer, then centrifuged at 3000 *g* for 20 min. The vacuolar zone was gently collected from the interface of the top and middle gradient layers. Vacuole enrichment was periodically checked under a microscope at 100× magnification; three fields were observed and criteria for preparation enrichment were: fewer than 4–5 non-lysed protoplasts in each field (15–40 vacuoles in each field) and no obvious contamination of other organelles. Then the vacuole preparations were frozen in liquid nitrogen and stored at -80°C for further use.

### SDS-PAGE and Western Blot of Total Vacuolar Proteins

As the amounts of protoplast and vacuole preparations were limited, the proteins in these preparations were initially quantified using the Bradford method (Bradford, 1976), and then stored at -20°C for further use. An aliquot (30 μl) of protoplast and vacuole preparation was added to 5 μl 6× SDS loading buffer and incubated in a 65°C water bath for 10 min. After centrifugation at 10,000 *g* for 2 min, 20 μl of supernatant was subjected to 12% SDS-PAGE (the protein contents of each protoplast and vacuole preparation from the five sampling dates loaded on the gel are indicated in Supplementary Figure 1A) and then blotted onto a polyvinylidene difluoride (PVDF) membrane (0.45 μm, Millipore, Bedford, MA, United States). After blocking with 5% (w/v) non-fat milk in PBST (10 mM Na_2_HPO_4_, 1.8 mM KH_2_PO_4_, pH 7.4, 140 mM NaCl, and 0.01% w/v Tween-20), the PVDF membranes were incubated for 1 h with one of the following primary antibodies: V-type ATPase (epsilon subunit of tonoplast H^+^-ATPase; tonoplast marker, 26–31 kDa; Agrisera, Vannas, Sweden, AS07 213, diluted 1:5000), AtpA (alpha subunit of ATP synthase, chloroplastic; chloroplast marker, 55 kDa; Agrisera AS08 304, 1:5000), P-type ATPase (plasma membrane H^+^-ATPase; plasma membrane marker, 100 kDa; Agrisera, AS07 260, 1:5000), BiP (lumenal-binding protein; endoplasmic reticulum [ER] lumen marker, 73.5–80 kDa; Agrisera, AS09 614, 1:2000), ARF1 (ADP-ribosylation factor 1; Golgi marker, 21 kDa; Agrisera AS08 325, 1:1000), AOX1/2 (plant alternative oxidase 1 and 2; mitochondrial marker, 36–40 kDa; Agrisera AS04 054, 1:1000). After three washes in PBST, the membranes were incubated in the secondary antibody (anti-rabbit for V-type ATPase, AtpA, P-type ATPase, ARF1, and AOX1/2, anti-chicken for BiP, coupled to horseradish peroxidase, ZSGB-BIO, Beijing, China). Horseradish peroxidase activity was detected using a Chemiluminescence Blotting Substrate kit (M5 Hiper ECL Western HRP Substrate, Mei5, Beijing, China) according to the manufacturer’s instructions, and chemiluminescence imaging on a Fusion Solo 4M systems (Vilber Lourmat, Marne-la-Vallée, France).

### SDS-PAGE and In-Gel Digestion of Vacuolar Proteins

Vacuole preparations (150 μl) were solubilized in lysis buffer (7 M urea, 2 M thiourea, 0.1% w/v CHAPS, pH 8); after vortexing, proteinase inhibitor cocktail in lysis buffer (1:50, v/v) was added, and the protein solution was incubated in an ultrasonic bath for 10 s in 1 s on/1 s off cycle. After centrifugation at 14,000 *g* for 30 min, the supernatant was carefully collected and frozen at -80°C. The Bradford method was applied for protein quantification (Bradford, 1976). For each sample and replicates, 5 μg of vacuolar protein was taken for 12% SDS-PAGE and stained with Coomassie Brilliant Blue. The in-gel trypsin digestion of protein for LC-MS/MS followed a previously described protocol (Shevchenko et al., 2006). Briefly, the gels (Supplementary Figure 2) were destained until the backgrounds were clear, and each lane of the gels (7 cm in length), representing one replicate, was excised and cut into pieces of approximately 1 mm^3^. After complete destaining and washing, the gel pieces were incubated overnight at 37°C for tryptic digestion.

### LC-MS/MS

The digested peptides were separated using nanoflow HPLC. Peptide mixtures were loaded onto a custom-designed C18 trap column (Acclaim PepMap100 column, 2 cm × 100 μm, C18, 5 μm) in solution A (0.1% v/v formic acid) and then separated with a custom-designed capillary C18 column (EASY-Spray column, 12 cm × 75 μm, C18, 3 μm) by a 90-min gradient from 4 to 95% acetonitrile in 0.1% formic acid at a flow rate of 350 nl/min. A Thermo Scientific Orbitrap Fusion Tribrid Mass Spectrometer was used for the mass analysis. The spray voltage of the EASY-Spray ion source was set to 2.1 kV. Survey scans were acquired with a resolution of 70,000 FWHM over 350–1800 m/z and the HCD spectrum resolution was 17,500 FWHM. The normalization collision energy was set to 29%.

### Database Search and Protein Identification

The raw MS data were analyzed using MaxQuant software suite (version 1.5.2.8^1^). The MS data were searched against the *V. vinifera* database in UniProtKB (29,907 entries, downloaded on October 20, 2017). For the initial search, the precursor mass window was 15 ppm. The search followed the enzymatic cleavage rule for trypsin; maximum two missed cleavage sites and 20 ppm mass tolerance for the fragment ions were allowed. For database searching, cysteine carbamidomethylation was considered a fixed modification, while N-terminal acetylation and methionine oxidation were considered variable modifications. The cutoff for the global false discovery rate (FDR) in the peptide-spectrum match (PSM) and protein identification was <0.01. Complete peptide and protein information from the triplicate analyses of the five samples is available in the ProteomeXchange (Vizcaino et al., 2014) Consortium via the PRIDE partner repository with the dataset identifier PXD010556, username: reviewer52388@ebi.ac.uk, password: Cbj9crka. Only proteins identified by at least two different peptides and in at least two replicates of a sample were regarded as present and reliably identified; these were subjected to further analysis.

### Protein Quantification and Data Processing

Label-free quantification (LFQ) of each identified protein was calculated using peptide signal intensities; the MaxLFQ algorithm in MaxQuant was activated to quantify protein abundance for the identified peptides as previously described (Cox and Mann, 2008; Cox et al., 2014). Match-between-runs was performed within the experimental replicates to extract the quantification information across the replicates (Cox et al., 2009). For low-abundance proteins with missing values, a value from the normal distribution with default parameters was complemented by Perseus (version 1.4.1.3) following Tyanova et al. (2016). Protein quantification and statistical significance analysis were performed using two-way Student’s *t*-test. Proteins that were quantified with arbitrary fold change (FC) of >1.5 and significance value of *P* < 0.05 on one sampling date compared to the other dates were designated as differentially abundant proteins (DAPs); log_2_ (fold change) was used for further analysis.

### Bioinformatics Analysis

Using the *V. vinifera* database in UniProtKB, we identified 2533 proteins; 1985 (78.4%) of them were annotated as putative uncharacterized proteins (Supplementary Data File 1: protein group list column B), and therefore the identified protein sequences were further blasted against the *A. thaliana* protein database in UniProtKB (39,211 entries, downloaded on July 18, 2017) with the BLASTP function in NCBI (v. 2.2.31^2^) for functional analysis. Gene ontology (GO) mapping and annotation of the identified proteins were conducted using Blast2GO Software (Bioinformatics Department, CIPF, Valencia, Spain^3^) (Götz et al., 2008). Heat maps and protein clustering were conducted by R^4^. The Venn diagram was drawn with Draw Venn Diagram^5^. Protein band intensities were quantified using Image J software (NIH^6^). Transport protein classification was according to TCDB^7^.

## Results

### Grape Berry Development and Vacuole Isolation

**FIGURE 1:**
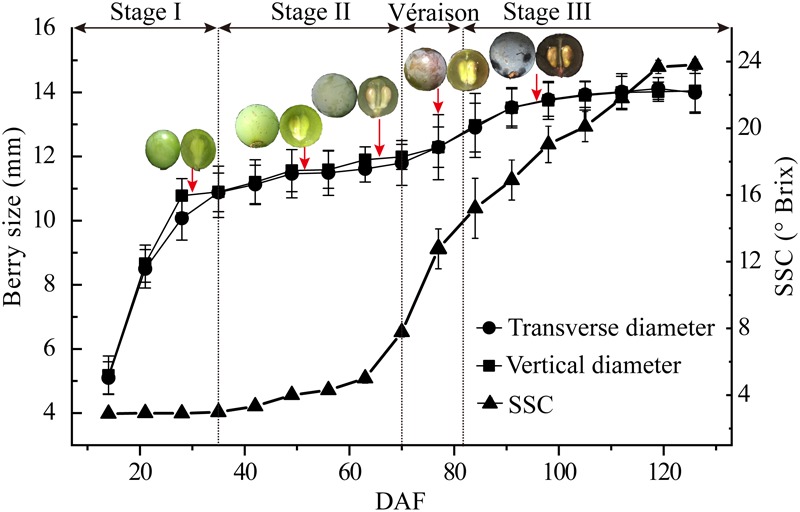
Major physiological parameters during grape berry development. Growth stages were determined by the change in berry transverse and vertical diameters and soluble solid contents (SSC) from early fruit set to full ripening [15–125 days after flowering (DAF)]. Berry sampling (DAF) for protoplast and vacuole preparations and proteome analysis are indicted by arrows, and typical berry appearance is shown. Values are means ± SD of three replicates with 50 berries each.

Berry size demonstrated a typical double sigmoid curve with very similar transverse and vertical diameter changes of the berries. A sharp increment was seen from berry set to about 35 DAF, a lag phase from 35 DAF to around 70 DAF, just before véraison when berry skin anthocyanin accumulation starts, and the second berry size increment, which was far less pronounced than that during stage I, occurred from 70 DAF to about 98 DAF (Figure 1). Soluble solids content, mainly hexoses and organic acids, was low in the early stage of berry development, then increased sharply from late stage II (63 DAF) until around 120 DAF (Figure 1).

**FIGURE 2:**
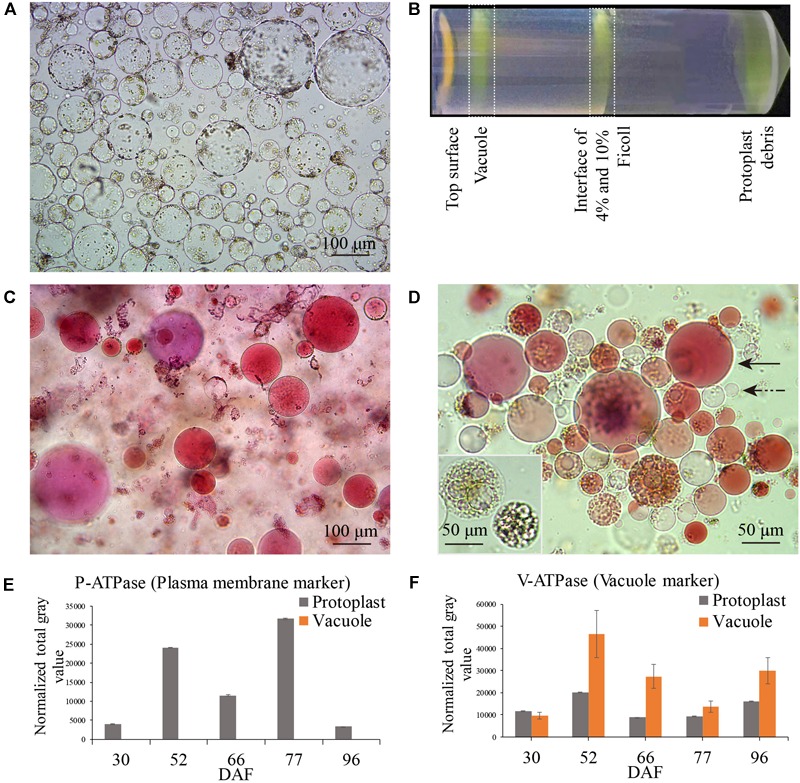
Grape berry flesh cell protoplast and vacuole preparations and quality check. **(A)** Isolated protoplasts. **(B)** Vacuole enrichment by Ficoll gradient centrifugation. **(C)** Crude vacuoles stained with Neutral Red. **(D)** Vacuoles in vacuole-enriched preparation stained with Neutral Red, solid arrow indicating vacuole, dashed arrow indicating tonoplast vesicle, inset indicating vacuoles with microdomains. **(E)** Relative quantity of P-ATPase between protoplast and vacuole isolations by western blotting analysis. **(F)** Relative quantity of V-ATPase between protoplast and vacuole isolations by western blotting analysis. Values were the average of three replicates ± SD.

Berry samples for vacuole isolation were collected on 30, 52, 66, 77, and 96 DAF, corresponding to late stage I, middle and late stage II, véraison and middle stage III, respectively (Figure 1, red arrows). The released protoplasts are shown in Figure 2A, and the vacuoles were further isolated by discontinuous Ficoll gradient centrifugation (Figure 2B). Vacuoles were intensely stained with Neutral Red in both crude and vacuole-enriched preparations (Figure 2C,D). Typical large central vacuoles were observed in our vacuole preparations, together with vacuoles with complex microdomains. The inset in Figure 2D shows vacuoles with complex microdomain in the vacuole preparation. The sizes of both protoplast and vacuole were heterogeneous, most of them ranging from 10 to 100 μm in diameter, but no typical representative size was observed.

The enrichment of the vacuole preparation for the proteomics study was first visually checked under a bright-field microscope, with criteria in three field replicates of fewer than 4–5 non-lysed protoplasts in each field of 15–40 vacuoles and no obvious contamination with other organelles. The vacuole preparations were further checked by western blotting using organelle-specific antibodies (Supplementary Figure 1A). As the actin content decreased markedly at véraison and stage III, the signal of the compartment-specific markers on the western blot was normalized to the amount of protein loaded in each lane. The relative activity of the plasma H^+^-ATPase (P-ATPase) and tonoplast/vacuolar H^+^-ATPase (V-ATPase) confirmed comparatively successful vacuole enrichment (Figure 2E,F), no signals of plasma membrane and chloroplast marker in vacuole preparations were detected, signals of ER, mitochondria, golgi were detected at low extent in vacuole preparations at different sampling dates (Supplementary Figure 1B).

### Berry Vacuole Proteome Landscape

**FIGURE 3:**
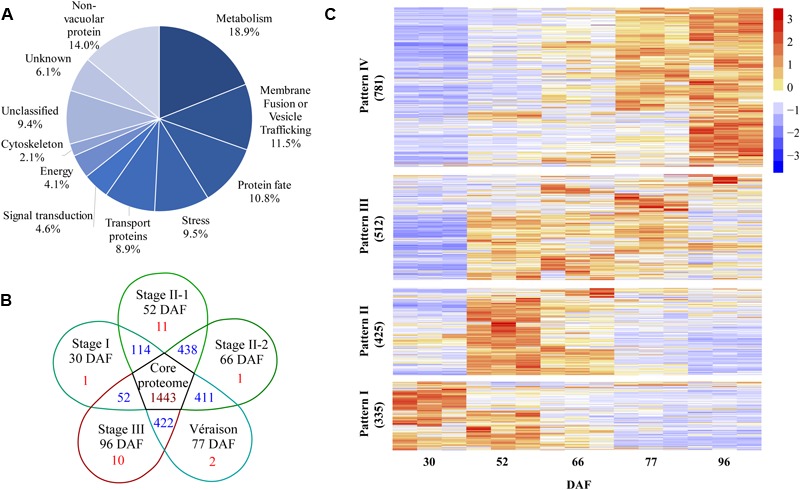
Global information on all quantified proteins from five samples taken during grape berry development. **(A)** Protein functional category distribution. **(B)** Venn diagram of identified proteins, core proteome is in the center. **(C)** Pattern of changes in protein abundance along berry development and ripening; values used for this analysis are normalized label-free quantification intensities. DAF, days after flowering.

A total of 2553 proteins were identified from the five vacuole samples and their replicates using the screening criteria of at least two different peptides identified and detected in at least two out of three biological replicates of a particular berry stage sample (Supplementary Data File 1 and Supplementary Table 1). All of the identified proteins were classified into 11 major categories based on GO annotations and the literature. The three largest groups were metabolism, membrane fusion/vesicle trafficking, and protein fate. Transport proteins was the fifth largest protein group with 8.9% of total identified proteins (Figure 3A). A set of 1443 proteins present in all berry vacuole preparations was regarded as the core proteome: there were 1, 11, 1, 2, and 10 unique proteins for 30 DAF (late stage I), 52 DAF (mid stage II), 66 DAF (late stage II), 77 DAF (véraison) and 96 DAF (stage III), and each pair of neighboring stages shared 114, 438, 411, and 422 proteins, respectively (Figure 3B).

After normalization of protein abundance in the three replicates from each of the five sampling dates, the changes in abundance of the identified vacuole proteins reflected a continuous change in the vacuole proteome landscape underlying berry development and ripening (Figure 3C). The change in abundance of vacuole proteins on the five sampling dates could be clustered into four major patterns. Pattern I was shown by 335 vacuole proteins: these were most abundant in late stage I to mid stage II, decreased in late stage II, and low abundance was seen from véraison to mid stage III. Pattern II, shown by 425 vacuole proteins, consisted of highest abundance in the middle of stage II, followed by late stage II, and low abundance in stages I and III. Pattern III, shown by 512 proteins, consisted of lowest abundance in late stage I, highest abundance in stage II and véraison, and low abundance in stage III. Pattern IV was shown by 781 proteins, the largest group: these proteins increased in abundance from stage I to stage II and reached peak abundance in stage III (Figure 3C and Supplementary Table 2).

### Expression Pattern of DAPs

**FIGURE 4:**
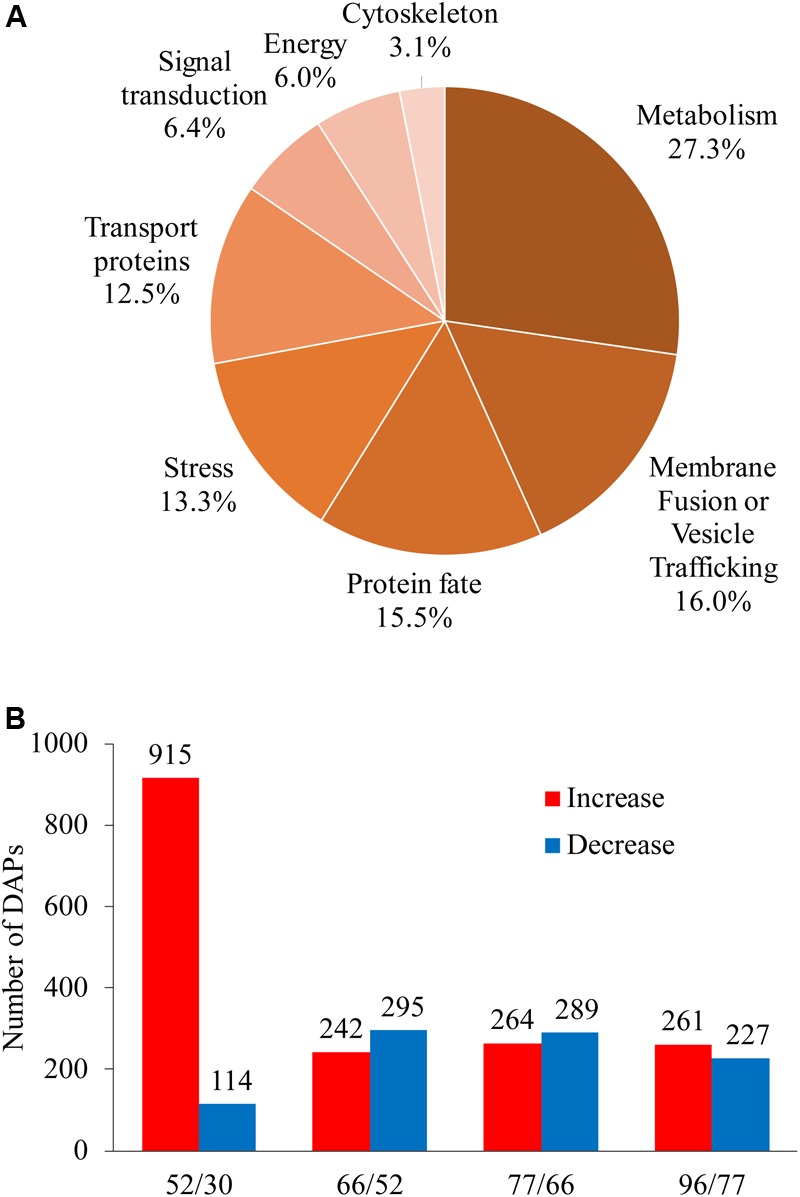
Global information on differentially abundant proteins (DAPs) identified from five sampling dates taken along grape berry development. **(A)** Functional category distribution of DAPs along grape berry development and ripening. **(B)** Increased and decreased DAP amounts in pairwise comparisons: 52/30, 66/30, 77/30, and 96/30 days after flowering. DAPs were recruited by ≥1.5-folds abundance change and *P* < 0.05.

Differentially abundant proteins showed a fold change in abundance of over 1.5 (*P* < 0.05) along berry development in comparisons between any two sampling dates; 1820 proteins were designated as DAPs, accounting for 88.6% of total identified proteins (Supplementary Table 3). Unclassified (167), unknown (96), and non-vacuolar (268) DAPs were discarded (531 proteins in total) in further analysis; the remaining DAPs (1289 proteins) were assigned to eight GO categories (Figure 4A). The largest category was metabolism, with 27.3% of the DAPs, followed by membrane fusion/vesicle trafficking (16.0%), protein fate (15.5%), stress response (13.3%), and transport proteins (12.5%); smaller groups were signal transduction (6.4%), energy (6.0%), and cytoskeleton (3.1%) (Figure 4A).

We focused on two landmark conversions during grape berry development: the first was from late stage I to mid stage II when berries enter the lag phase of growth, and the second was from late stage II to véraison, when berries begin to rapidly accumulate sugar along with other changes related to quality formation. The numbers of increased and decreased DAPs for berries in late stage I to mid stage II (52 vs. 30 DAF), late to middle stage II (66 vs. 52 DAF), late stage II to véraison (77 vs. 66 DAF), and véraison to ripening (96 vs. 77 DAF) were 915 and 114, 242 and 295, 264 and 289, and 261 and 227, respectively (Figure 4B).

A very pronounced proteomic change was revealed between late stage I and mid stage II, characterized by high numbers of DAPs and a high ratio of increased to decreased abundance DAPs. GO categories of transport proteins, energy, and signal transduction demonstrated the highest increase-to-decrease ratio: 96/6, 44/2, and 42/6, respectively. From mid stage II to véraison, in agreement with the slower berry growth, more proteins with decreased abundance were found for the energy, metabolism, stress, and cytoskeleton categories. From véraison to ripening, increased abundance was mainly seen for energy and protein-fate proteins, whereas similar numbers of increments and decreases were found for transport proteins, signal transduction, metabolism, stress, and membrane fusion/vesicle trafficking category proteins, and a strong decrease was found for cytoskeleton proteins (Table 1).

**Table 1:** Number of increased and decreased differentially abundant proteins (DAPs) in eight functional categories in comparisons between adjacent sampling dates^a^.

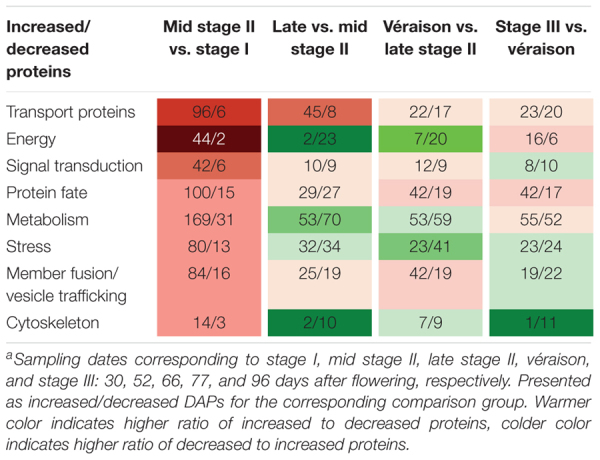

### Transport Proteins

**FIGURE 5:**
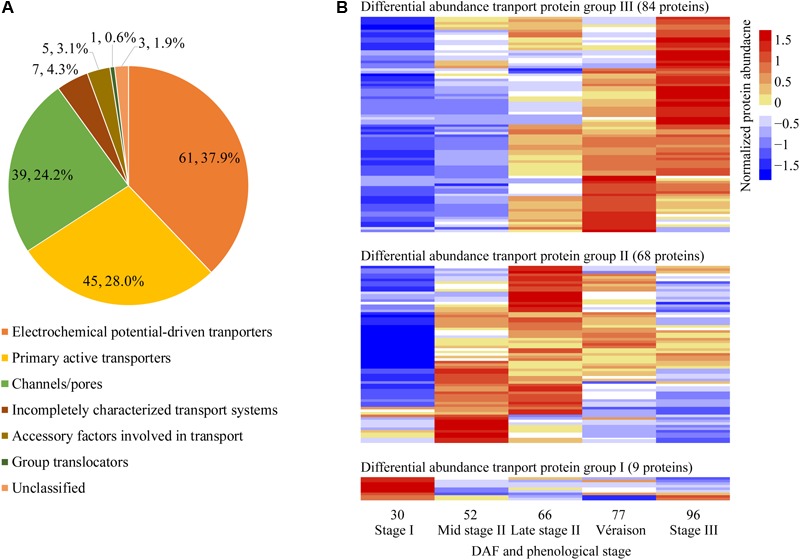
Classification and pattern of changes in abundance of differentially abundant transport proteins along berry development and ripening. **(A)** Classification of differentially abundant transport proteins identified from five sampling dates taken along grape berry development according to TCDB (http://www.tcdb.org/). **(B)** Differentially abundant transport protein group; Normalized protein abundance; DAF, days after flowering.

With pronounced increments in berry weight and soluble solids content from late stage II to berry ripening, the vacuole transport proteins were of special interest in the present study; 161 transport proteins annotated as localized to the vacuole and other potentially contaminating organelles exhibited changes in differential abundance along berry development (Supplementary Data File 2). Differentially abundant transport proteins were classified into seven categories according to TCDB^8^: 61 electrochemical potential-driven transporters, 45 primary active transporters, 39 channels/pores, 7 incompletely characterized transport systems, 5 accessory factors involved in transport proteins, 1 group translocator and 3 unclassified transport proteins (Figure 5A). These transport proteins could be further classified into 62 families (Supplementary Data File 2). Most of the differentially abundant transport proteins exhibited their highest abundance at the berry ripening stages; 9, 68, and 84 were most abundant at stage I, stage II, and véraison/stage III, respectively (Figure 5B). Grape berry is a fleshy fruit, and water, sugars, acids and secondary metabolism constituents, such as pigments, are major contributors to fruit yield and quality. Therefore, vacuolar proteins involved in transporting these substances, i.e., proton pumps, aquaporins, sugar transporters, major facilitator superfamily (MFS) proteins, ion transport proteins, ATP-binding cassette (ABC) transporters, and amino acid and peptide transport proteins, were of highest interest, and selected to elucidate their abundance changes during grape berry quality formation in the present study.

#### Proton Pumps

**FIGURE 6:**
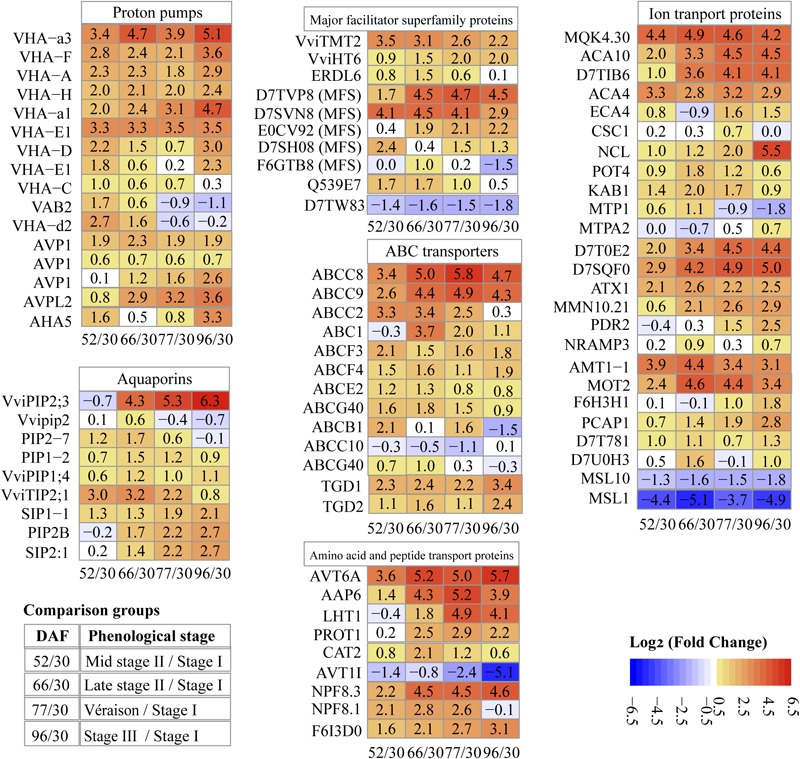
Pattern of changes in abundance of key transport proteins along berry development and ripening. Values in each block are log_2_ fold change. 52/30, 66/30, 77/30, 96/30, pairwise comparisons of sampling points (DAF, days after flowering).

Transport across the tonoplast is energized by two types of proton pumps, V-ATPase and V-PPase (vacuolar H^+^-pyrophosphatases). V-ATPase contains 14 distinct functional subunits (Marshansky et al., 2014); we detected 11 subunits, the most V-ATPase subunits quantified in grape proteomes reported to date; 4 and 7 of them were most abundant at stage II and véraison/stage III, respectively (Supplementary Data File 2). All of the V-ATPase subunits were more abundant at mid stage II (52 DAF) than at stage I (30 DAF), with a log_2_ FC value of 1–3.3; the rate of abundance increase for all subunits decreased during stage II, with subunits B2 and d2 decreased by 1.1–0.2 log_2_ fold at ripening (Figure 6). The results were generally in line with a study using grape berry tonoplast vesicles as the study material that showed increasing V-ATPase during grape development, a slight drop in abundance around véraison, and another increase during ripening (Terrier et al., 2001).

H^+^-PPase consists of a single polypeptide that uses the energy from pyrophosphate hydrolysis to drive proton translocation across membranes (Maeshima, 2001; Martinoia et al., 2007). Two types of H^+^-PPases, type I and type II, have been reported in many species: type I (AVP1) is located in the tonoplast, and type II (AVPL2) is located in the Golgi apparatus and the trans-Golgi network (Segami et al., 2010). In our study, three isoforms of type I and one type II enzyme were identified (Figure 6). One AVP1 (D7UA22) was markedly more abundant than the other H^+^-PPases, 8.4, 12.7, 13.6, 12.3, and 13.4 for late stage I (Supplementary Data File 2), middle and late stage II, véraison and ripening, respectively. The second AVP1 (D7T4X2) maintained its abundance increment across the sampling dates with double the amount at ripening compared to véraison. The third AVP1 (D7TZ79) demonstrated highest abundance in late stage II, with around 3/4 of peak abundance at véraison and berry ripening. AVPL2 (D7SS21) continuously increased during grape berry development and ripening (Figure 6). Although the predicted localization of AVPL2 was the Golgi apparatus, it has also been identified and reported in vacuole proteomes (Shimaoka et al., 2004; Schmidt et al., 2007). V-PPase has been reported as the predominant proton pump in intact vacuoles of ripening grape isolated from berry suspension cultured cells (Fontes et al., 2010a) and grape berry tonoplast vesicles (Terrier and Romieu, 1998; Terrier et al., 2001). Repression of V-PPase activities has been reported during tomato and pear ripening (Milner et al., 1995; Shiratake et al., 1997).

#### Aquaporins

Aquaporins are members of the major intrinsic protein (MIP) family, implicated in water transport across biological membranes; they may also play a role in transporting small solutes (Gomes et al., 2009). In our study, one differentially abundant aquaporin was annotated as a tonoplast intrinsic protein (TIP), six as plasma membrane intrinsic proteins (PIPs), and two as small and basic intrinsic proteins (SIPs). Six of them were previously reported by other vacuole or tonoplast proteomic studies (Supplementary Data File 2). Along berry development, five aquaporin proteins exhibited highest abundance at stage II, and four at stage III (Figure 6 and Supplementary Data File 2).

The grapevine genome predicts 10 TIP-encoding genes, but only three of them, i.e., VviTIP1;2, VviTIP2;1, and VviTIP1;1, have been confirmed in grape berries at various developmental stages (Fouquet et al., 2008; Vandeleur et al., 2009). In our results, one TIP (VviTIP2;1) was identified, with specific protein abundance values of 29.3, 230, 266, 132.6, and 50.4 on the five chronological sampling dates, respectively (Supplementary Data File 2); its abundance peaked at the end of stage II (66 DAF), and decreased during véraison and ripening (stage III), exhibiting both the most intense increment and decrease among MIP members identified in this study (Figure 6). Its pattern of change in protein abundance was in agreement with earlier reports in peach and grape (Shiratake et al., 1997; Fouquet et al., 2008).

#### MFS Proteins

The vacuole serves as the main reservoir for sugars, and tonoplast transporters play critical roles in sugar accumulation and remobilization. Ten DAPs were found to be members of the MFS, all of them previously identified in other vacuolar proteomic studies; 1, 5, and 4 of them showed peak abundance at stage I, stage II, and véraison/stage III, respectively (Supplementary Data File 2). Among them, 3 differentially abundant sugar transport proteins were identified, i.e., VviTMT2 (tonoplast monosaccharide transporter, TMT2, now renamed TST), VviHT6 (hexose transporter 6), and ERDL6 (early response to dehydration like 6) (Figure 6 and Supplementary Data File 2). The abundance of VviTMT2 increased 3.5 log_2_ fold in mid stage II, whereas the abundance of VviHT6 increased continuously with berry development and maintained high abundance during ripening; the protein abundance of ERDL6 peaked at late stage II, and decreased at véraison and stage III (Figure 6).

Five MFS proteins were differentially abundant; three of them exhibited highest abundance at véraison/stage III, and the other two at late stage II (Figure 6 and Supplementary Data File 2). Regardless of the specific changes in protein abundance along the five sampling dates, all of the mentioned sugar transport and MFS proteins (except for F6GTB8) exhibited higher abundance at stage III compared to stage I (Figure 6 and Supplementary Data File 2).

#### ABC Transporters

ABC transporters are a large family that functions in translocating multiple substrates across the membrane, often against the concentration gradient powered by ATP (Theodoulou, 2000). Thirteen ABC transporters belonging to six different subfamilies were DAPs, with 7 of them having been reported in at least one previous vacuole or tonoplast proteome study (Supplementary Data File 2); 1, 9, and 3 ABC transporters exhibited their highest abundance at stage I, II, and véraison/stage III, respectively (Supplementary Data File 2).

Among the ABC transporters, the ABCC subfamily contains vacuole-localized proteins mediating transportation of glutathione and glucuronate conjugates in yeast, *Arabidopsis* and other plant species (Martinoia et al., 2012). Four ABCC proteins (formerly named multidrug resistance proteins) were identified in our study; the abundance of ABCC2, ABCC8, and ABCC9 largely increased with berry development, 3.3, 3.4, and 2.6 log_2_ fold, respectively, in mid stage II compared to stage I; the abundance of ABCC8 and ABCC9 increased sequentially 1.6 and 1.8 log_2_ fold, respectively, in late vs. mid stage II. The abundance of ABCC8 at véraison was 5.8 log_2_ fold higher than that at stage I (Figure 6). Both ABCC8 and ABCC9 decreased after véraison.

#### Other Transport Proteins

Another 113 DAPs were annotated as transport proteins of: ions (25 proteins), amino acids and peptides (9 proteins), nucleobases (3 proteins), plant hormones (4 proteins), lipids (1 proteins), and other substrates (71 proteins).

The 25 differentially abundant ion transport proteins included 17 metal, 2 anion, 1 cation, and 5 other unknown ion transport proteins (Figure 6 and Supplementary Data File 2); 14 of them were clustered in differentially abundant transport protein group III, and exhibited their highest abundance at stage III (Supplementary Data File 2). The differentially abundant metal ion transport proteins included 6 calcium, 1 sodium/calcium, 2 potassium, 2 zinc, 1 tellurium, 1 magnesium, 1 copper, 1 sodium, 1 manganese, and 1 metal ion transport proteins; the log_2_ FC values of these transport proteins are shown in Figure 6. The two anion and one cation transport proteins were related to ammonium, molybdate, and chloride ion transport (Figure 6).

Apart from the transport proteins for inorganic compounds, several organic compound transport proteins were also quantified in the present study. Amino acid and peptide transport proteins were the most abundant of this type, with 1, 2, and 6 DAPs having their highest abundance at stage I, stage II, and the ripening stages (véraison/stage III), respectively (Figure 6 and Supplementary Data File 2).

## Discussion

Vacuole size and contents changed dynamically during grape berry development and ripening. As the largest compartment of grape berry flesh cells, the vacuole plays a central role in berry development and quality formation. However, information on the proteomic base of these changes was lacking, mainly due to the difficulty involved in isolating vacuoles. Using a modified protocol, grape mesocarp cell vacuoles from five sampling dates covering all three grape berry developmental stages were isolated and subjected to comparative quantitative proteomics analysis. Our results provide the first global view of grape berry vacuole proteins’ changing patterns, including those of transport proteins.

### Validation of Vacuole Isolation and Enrichment

The vacuole is bounded by a single phospholipid bilayer, the tonoplast, making it more fragile than other organelles with two phospholipid bilayers. The versatile vacuole is essential to plant growth and responses to biotic and abiotic stress, but knowledge of its protein population is still lacking, the main barrier being the difficulty in isolating vacuoles and tonoplasts for most plant species. Intact vacuoles have been isolated from *Arabidopsis* leaf and suspension cells, barley leaf cells, cauliflower buds, sugar beet taproot cells and ripe grape berry mesocarp suspension cells. The complex composition and high concentration of contents in the fleshy fruit vacuole lumen make vacuole isolation more difficult than for other plant tissues.

In our experiments, a previous protocol (Fontes et al., 2010a,b) was adopted and modified. We increased the concentrations of cellulose and pectinase from the previously reported 0.03% (w/v) and 0.003% (w/v), respectively, to 1.5% (w/v) and 0.3% (w/v), respectively, eliminated the use of plant hormones, and strengthened the washing step before protoplast isolation, a critical step. Moreover, we shortened the time for enzyme digestion from 12 h to 45 min, because less time for cell wall digestion during protoplast isolation may better retain the protoplasts’ original state as in plant cells (Ohnishi et al., 2018). More importantly, this modified vacuole isolation protocol was also applicable for our experiments with berries of *Vitis davidii* (data not shown), which is a low-sugar-accumulating *Vitis* species, and with a few more adjustments based on the present study, we also successfully isolated protoplasts and vacuoles of fig female flower tissue. This suggests that the methods in the present study may applicable to other fleshy fruit.

The enrichment of the isolated vacuoles was confirmed and validated by morphological observation, biochemical testing and annotation of protein types. We stained the crude vacuoles and those in the vacuole-enriched preparation with Neutral Red; the intense red color of the vacuoles suggested their integrity, due to well-maintained internal acidic pH and the activity of proton pumps. There were obvious size-distribution differences among protoplasts and vacuoles isolated from different samples. Such heterogeneity has been previously reported with vacuoles isolated from grape mesocarp suspension cells (Fontes et al., 2010b). Due to the change in turgor and contents of the vacuoles as the berries develop, the amount of isolated vacuolar content was different on different sampling dates; in general, the yields of protoplasts and vacuoles at late stage II (66 DAF) and véraison (77 DAF) were lower than on the other sampling dates.

Occasionally, non-lysed protoplasts, plasma membrane fragments attached to the vacuoles and a few chloroplasts were observed under the microscope, as previously reported in grape berry subepidermal cell vacuole isolation, where the attached plasma membrane could not be removed (Moskowitz and Hrazdina, 1981). Microdomains have been reported in yeast cell, *Arabidopsis* cultured cell and sugar beet root cell vacuolar membranes (Toulmay and Prinz, 2013; Yoshida et al., 2013; Cui et al., 2016). Grape berry vacuoles with microdomains were much more prevalent at stage III than at the other stages in our vacuole preparations (a typical image of a vacuole with microdomains is shown in the inset to Figure 2D). Moreover, a complex structure of the grape berry mesocarp vacuole was observed, with the reported typical morphological traits of the “vacuolar apparatus” (Fontes et al., 2010b) – “bulb” (Saito et al., 2002), intravacuolar sheets in the vacuole lumen (Uemura et al., 2002), tubular vacuoles (Hicks et al., 2004), and main vacuoles with many small vesicles attached (Shimaoka et al., 2004).

Biochemically, the overall quality of the vacuole isolation was supported by western blotting with anti-P-ATPase and anti-V-ATPase antibodies. The vacuole marker epsilon subunit of tonoplast H^+^-ATPase (V-ATPase) was highly enriched in the vacuole preparation compared to its corresponding protoplast preparation, and no or extremely low P-ATPase signal was detected in the vacuole preparations. The signals of the ER, mitochondria, Golgi, and chloroplast markers were detected to different extents in the vacuole preparations (Figure 2E,F and Supplementary Figure 1B). Signals for the mitochondrial marker (AOX1/2, 52, 66, and 96 DAF) and Golgi marker (ARF, 52 and 77 DAF) were high in vacuole samples, indicating possible antigen enrichment resulting from contamination in the isolated vacuole preparation. Proteins are transported into vacuoles for degradation (De, 2000), and entire organelles, such as mitochondria, plastids and damaged chloroplast, have been observed engulfed by vacuoles for autophagy (Carter et al., 2004; Izumi et al., 2017). Moreover, the high ER marker (BiP) signal in the vacuole peaked at 96 DAF, indicating that there could be a marked increase in vacuolar protein transported to the vacuole via ER bodies during berry ripening. The presumed contamination could be the result of the vacuole’s protein-lysis function, or to the differences in the amount of protein loaded in each lane for the western blotting, although differences in contamination levels among samples cannot be excluded.

Annotation results also supported enrichment of the isolated vacuoles; 14.7% (268/1820) of the DAPs were annotated as non-vacuolar proteins, which were mainly predicted as chloroplast and mitochondrial proteins without predicted transmembrane domains, and these were excluded from further analysis; specifically, 14.5, 14.2, 14, 13.6, and 13.9% non-vacuolar DAPs were revealed for the five sampling dates, respectively, which was generally in line with the western blotting validation using markers of other organelles and contamination levels observed in photographs of the vacuole fraction. Cross-contamination with membranes from other compartments is inevitable in proteome analyses; the vacuole is the terminal location for various endomembrane trafficking pathways, and expansion of the tonoplast and contents in the vacuole lumen occurs from different sources (Krüger and Schumacher, 2018). The presence of non-vacuolar proteins in the vacuole preparations could be a consequence of autophagy (Li and Vierstra, 2012), direct ER-to-vacuole trafficking (Rojo et al., 2003; Cui et al., 2016), internalization of cytosolic proteins or engulfment of various cellular components (Carter et al., 2004; Izumi et al., 2017), which certainly could be identified by the proteomic studies. For those important proteins that have never been validated by localization, confirmation of subcellular compartmentalization beyond proteomics studies is suggested to further elucidate the mechanism of key biological processes.

### Changes in the Vacuole Proteome for New Storage Requirements and Routine Functions

Compared to previous vacuole proteome reports using mesophyll and other vegetative organ cells, our study used a sink tissue and covered a long period during which the berry flesh cells experience remarkable physiological and functional changes; with respect to the vacuoles, they started as vacuoles similar to those of vegetative tissues which do not accumulate high amounts of sugar, and ended up as high-sugar-containing organelles. Grape berry growth and quality formation have been well studied (Coombe, 1992). At véraison, the berries change in color and texture, accumulate sugars and develop flavors. Along with fundamental and significant changes in berry cell content during stage III, the most active change in protein expression was expected at véraison. Experimentally, a whole-cell soluble proteome study using table grape berry (*V. vinifera* L. cv. Muscat Hamburg) reported two key developmental transitions: from stage I (7 mm) to stage II (15 mm), and from stage II (15 mm) to véraison; numbers of DAPs and log_2_ fold changes in maximum/minimum protein abundance were larger in the second transition (Martínez-Esteso et al., 2013). However, we found this not to be the case for wine grape vacuoles.

In our study, the most substantial changes in the vacuole proteome were found between 30 DAF and 52 DAF, i.e., late stage I to mid stage II, when berry size increases slowly (Figure 3C, 4B). The 915 DAPs that increased and the 114 DAPs that decreased were distributed among the GO categories of metabolism, protein fate, transport proteins, membrane fusion/vesicle trafficking, stress, signal transduction, energy and cytoskeleton. All of the categories had more DAPs during this conversion than during the conversions from late stage II to véraison or from véraison to mid stage III, the latter two periods standing out far more prominently both phenotypically and physiologically. Thus a massive vacuole proteome event during the quieter period of berry performance was uncovered.

The reason for the lag phase in berry development at stage II has long been a mystery. As seedless cultivars have a shorter stage II, the lag phase was suggested to be required for seed development, when the seed embryo develops with a concomitant hardening of the seed coat (Kennedy et al., 2000). Nevertheless, the existence of a lag phase in seedless berries implies that this rather steady period of berry development could be fulfilling other needs. Our proteome results revealed substantial changes in the protein population of the vacuole – the largest organelle in berry cells – during this stage.

The modified vacuole properties in the early stage of berry development could be confirmed by the functions of the DAPs during this conversion: a number of glycosidases, protein degradation, stress response, membrane fusion and remodeling proteins, which have been reported capable of affecting tonoplast physicochemical properties (Carter et al., 2004). This is also in line with high accumulation of organic acid during the lag phase (Coombe, 1992). Similar to previous reports, another significant change in the vacuole’s protein landscape was found from véraison to ripening (Martínez-Esteso et al., 2013). A very high abundance increment was seen for DAPs categorized as V-ATPases, ion transport proteins, sugar transporters, ABC transporters, MFS proteins, and others at berry ripening, indicating the vacuole proteome’s conversion toward the dramatic increase in vacuole lumen contents of sugars and other berry quality-related substances.

In the late stage of berry development, the flesh cell vacuole can be regarded as a highly functional specialized container for soluble carbohydrates; together with the biological importance of sugars, sugar transporters have been focused on in plant vacuole studies, even those studying vegetative tissue of model plants (Schulze et al., 2012). However, it is worth remembering that the primary biological functions of the vacuole are lysis and degradation in lytic vacuoles, and storage in protein storage vacuoles. The large central vacuoles present in mature vegetative cells are considered lytic (Carter et al., 2004; Jaquinod et al., 2007). Our results show maintenance of the lytic function in a late stage of berry development when a high concentration of sugars is accumulated in the vacuole. The largest proportion of DAPs were in the metabolism category; 352 proteins (27.2% of the DAPs) belonged to the subcategories of amino acid metabolism, lipid and fatty acid metabolism, glycosidase and others, while the protein-fate category included 200 proteins (15.5% of total DAPs); more than half of these (115 DAPs) were annotated to the protein degradation subcategory, and the amounts were steady and continuously comprised the largest category along berry development (Supplementary Figure 3). From véraison to berry ripening, increments of amino acid and peptide transport proteins, nucleobase transport proteins, ER proteins and others supported our hypothesis that berry flesh vacuoles take on the new function of sugar accumulation in the late stage of berry development and in parallel, retain their primary and typical lytic functions.

### New Findings With Sugar Transporters

In the present study, three sugar transporters – VviHT6, VviTMT2, ERDL6 – were identified as DAPs. All of them were noteworthy for being recorded for the first time in the grape proteome at the vacuole and/or tonoplast level. Six VviHTs – VviHT1–6 – have been identified in grape berries: VviHT1, VviHT4, and VviHT5 with confirmed plasma membrane localization, and VviHT2 and VviHT6 with suggested tonoplast localization (Bordenave et al., 2013). The tonoplast localization of VviHT6 was further predicted by its high sequence similarity with the tonoplast transporter of *Arabidopsis* AtTMT2 (Afoufa-Bastien et al., 2010). The transcript of VviHT6 peaks at véraison, and the protein has been suggested to be responsible for hexose accumulation in the vacuole at the onset of ripening (Conde et al., 2006; Deluc et al., 2007). In our study, protein abundance of VviHT6 was 3.0, 5.5, 8.2, 11.8, and 12.1 in late stage I, the two sampling points of stage II, véraison and the middle of stage III, respectively. The continuous increase in protein abundance provides new evidence for the potentially important role of VviHT6 in berry vacuole sugar accumulation.

In grape berry, the TMTs were VviTMT1, VviTMT2, and VviTMT3. VviTMT1 was localized to the tonoplast in our previous study (Zeng et al., 2011), and is the only grape tonoplast monosaccharide transporter to be localized and functionally studied. In addition to the present vacuole proteome identification, the tonoplast positioning of VviTMT2 is supported by its homologs in *Arabidopsis* (AtTMT2), sugar beet taproot (BvTST2), and sweet melon (CmTST2), all of which have been confirmed as tonoplast-located sugar transporters (Wormit et al., 2006; Jung et al., 2015). In our study, the abundance of VviTMT2 was 0.5, 5.7, 4.6, 3.1, and 2.3 on the five sampling dates, respectively, in good agreement with its transcript expression pattern (Cakir and Giachino, 2012).

ERDL6 is a tonoplast glucose proton symporter that delivers glucose from the vacuole to the cytosol in *Arabidopsis* (AtERDL6) and sugar beet (BvIMP) (Poschet et al., 2011). The transcripts of strawberry *ERDL6* (*FaSug10*) consistently increase during strawberry ripening in high-sugar-content cultivars, but a contrasting expression pattern has been shown in cultivars with low sugar content (Shanmugam et al., 2017); 6 *ERDL6* isoforms were found to be upregulated during sweet orange ripening, and it was speculated to play an important role in balancing the disruption of sugar contents in vacuoles caused by developmental changes (Klemens et al., 2013). The grape genome harbors 22 *ERD6-like* genes, none of whose expression or function has been investigated. In the present study, a grape ERDL6 was identified for the first time in grape berry vacuoles with decreasing abundance from véraison, when the accumulation of sugars commences, suggesting that grape ERDL6 may responsible for reducing glucose export from the vacuole to the cytosol, thus helping in glucose accumulation in ripening berries.

In summary, our results provide a first sketch of the vacuole proteome landscape as grape berries grow and ripen. The finding of a core proteome revealed regular tonoplast residents and vacuolar protein population, and function classification of the core proteome suggested that the routine lytic function of vacuoles in vegetative tissues is maintained in the central vacuole of berry flesh cells throughout grape development. The capacity for accumulation of high amounts of sugar and other contents during berry ripening could be regarded as an additional function operating in the late stage of vacuole development. A proteome surge was detected from late stage I to the middle of stage II, revealing a significant proteome conversion during the first transition of grape berry development, and providing new information on the biological changes underlying the phenotypical lag phase. Most of the proton pumps and other transport proteins increased in abundance until berry ripening, providing further evidence of a function for late-stage metabolite accumulation. Our results thus demonstrate the first grape berry vacuole proteome and its changing pattern during berry development and ripening.

## Author Contributions

LK, SC, and HM designed the experiments and analyzed the results. LK conducted the experiments. LK, SC, YG, and HM prepared the manuscript. All authors read and approved the manuscript for publication.

## Conflict of Interest Statement

The authors declare that the research was conducted in the absence of any commercial or financial relationships that could be construed as a potential conflict of interest.

## Footnotes

1 www.coxdocs.org

2 ftp://ftp.ncbi.nlm.nih.gov/blast/executables/blast+/LATEST/

3 https://www.blast2go.com/

4 https://www.r-project.org/

5 http://bioinformatics.psb.ugent.be/webtools/Venn/

6 http://rsbweb.nih.gov/ij

7 http://www.tcdb.org/

8 http://www.tcdb.org/
