# Supplementary material for: Quantitative Proteome Analysis Reveals Changes in the Protein Landscape During Grape Berry Development With a Focus on Vacuolar Transport Proteins
Source: Front Plant Sci. 2019 May 15;10:641. doi: 10.3389/fpls.2019.00641 (PMC6530609; doi:10.3389/fpls.2019.00641)
Supplement: Supplementary file 1 [file Table_1.docx]

**Supplementary Data**

**Quantitative Proteome Analysis Reveals Changes in the Protein Landscape during Grape Berry Development with a Focus on Vacuolar Transport Proteins**

Liuqing Kuang^1^, Shangwu Chen^2^, Yan Guo^3^, Huiqin Ma^1*^

^1^ Department of Fruit Tree Sciences, College of Horticulture, China Agricultural University, Beijing 100193, China

^2^ College of Food Science and Nutrition Engineering, China Agricultural University, Beijing 100193, China

^3^ College of Biology Sciences, China Agricultural University, Beijing 100193, China

Liuqing Kuang: [kuangliuqing@cau.edu.cn](mailto:kuangliuqing@cau.edu.cn)

Shangwu Chen: [swchen@cau.edu.cn](mailto:swchen@cau.edu.cn)

Guo Yan: [guoyan@cau.edu.cn](mailto:guoyan@cau.edu.cn)

Huiqin Ma: [hqma@cau.edu.cn](mailto:hqma@cau.edu.cn), *Corresponding author, telephone number: 86-10-62733986

**Supplementary Table 1** Number of proteins identified in each replicate and in the differentially abundant protein (DAP) filtration process.

|  | 30DAF | 52DAF | 66DAF | 77DAF | 96DAF |
| --- | --- | --- | --- | --- | --- |
| Replicate 1 | 1453 | 2078 | 2010 | 2035 | 2002 |
| Replicate 2 | 1452 | 2040 | 2013 | 2049 | 2051 |
| Replicate 3 | 1420 | 2095 | 1967 | 1962 | 2024 |
| Sum of 3 replicates | 1717 | 2260 | 2224 | 2230 | 2242 |
| Sum of 5 samples | 2533 | | | | |
| Quantified proteins | 2487 | | | | |
| Filtered by ≥2 peptides (for each sampling date) | 1578 | 1956 | 1949 | 1929 | 1937 |
| Filtered by ≥2 peptides (sum) | 2075 | | | | |
| Filtered by ≥2 replicates in one sample | 2053 | | | | |
| DAPs^a^ | 1820 | | | | |

^a^DAPs were recruited by fold change ≥1.5 and *P* < 0.05.

DAF, days after flowering.

**Supplementary Table 2** Functional category distribution of all identified proteins in four changing-abundance patterns along grape berry development and ripening.

| **Functional categories** | **Pattern I** | **Pattern II** | **Pattern III** | **Pattern IV** | **Total** |
| --- | --- | --- | --- | --- | --- |
| Metabolism | 72 | 92 | 81 | 144 | 389 |
| Membrane fusion/vesicle trafficking | 38 | 34 | 71 | 93 | 236 |
| Protein fate | 29 | 40 | 52 | 101 | 222 |
| Stress | 38 | 51 | 42 | 65 | 196 |
| Transport proteins | 14 | 28 | 59 | 81 | 182 |
| Signal transduction | 14 | 10 | 24 | 47 | 95 |
| Energy | 15 | 24 | 17 | 29 | 85 |
| Cytoskeleton | 15 | 11 | 6 | 12 | 44 |
| Unknown | 23 | 19 | 40 | 43 | 125 |
| Unclassified | 24 | 31 | 47 | 90 | 192 |
| Non-vacuolar proteins | 53 | 85 | 73 | 76 | 287 |
| **Sum** | **335** | **425** | **512** | **781** | **2053** |

Warmer color indicates higher amount of protein in corresponding group.

**Supplementary Table** **3** Functional category distribution of all identified proteins and differentially abundant proteins (DAPs) from five samples along grape berry development.

| **Functional category** | **All identified proteins** | | **DAPs** | |
| --- | --- | --- | --- | --- |
|  | Number | Percentage | Number | Percentage |
| Metabolism | 389 | 18.9% | 352 | 19.3% |
| Membrane fusion/vesicle trafficking | 236 | 11.5% | 206 | 11.3% |
| Protein fate | 222 | 10.8% | 200 | 11.0% |
| Stress | 196 | 9.5% | 171 | 9.4% |
| Transport proteins | 182 | 8.9% | 161 | 8.8% |
| Signal transduction | 95 | 4.5% | 82 | 4.5% |
| Energy | 85 | 4.1% | 77 | 4.2% |
| Cytoskeleton | 44 | 2.1% | 40 | 2.2% |
| Unclassified | 192 | 9.4% | 167 | 9.2% |
| Unknown | 125 | 6.1% | 96 | 5.3% |
| Non-vacuolar proteins | 287 | 14.0% | 268 | 14.7% |
| **Total** | **2053** |  | **1820** |  |

**Supplementary Figures**

**Supplementary Figure legends**

**Supplementary Figure 1** **Western blotting results for vacuole isolation quality control. (A)** Western blot. Protoplast (0.8–1.4 μg/lane) and vacuole (0.5–1.1 μg) proteins from five sampling dates (DAF) were subjected to 12% SDS-PAGE. Antibodies to actin (45 kDa), P-type ATPase (100 kDa, plasma membrane H^+^-ATPase, protoplast marker), V-type ATPase (26–31 kDa, epsilon subunit of tonoplast H^+^-ATPase, vacuole marker), BiP (80 kDa, lumenal-binding protein, ER marker), AOX 1/2 (36–40 kDa, plant alternative oxidase 1 and 2, mitochondrial marker), ARF1 (21 kDa, ADP-ribosylation factor 1, Golgi marker), and AtpA (55 kDa, alpha subunit of ATP synthase, chloroplast marker) were used. **(B)** Relative quantities of BiP, AOX1/2, ARF1 and AtpA between protoplast and vacuole isolations. Protein band intensities were quantified using Image J software (NIH, http://rsbweb. nih.gov/ij), and normalization to the corresponding protein content. Values are averages of three replicates ± SD. DAF, days after flowering.

**Supplementary Figure 2** Representative SDS-PAGE of vacuole proteins from grape berry mesocarp. Vacuole proteins in samples taken 66, 77, and 96 days after flowering are presented. Black boxes indicate lane excision for further analysis.

**Supplementary Figure 3** **Subcategory distribution of differentially abundant proteins (DAPs) into metabolism and protein fate. (A)** Metabolism DAPs. **(B)** Protein-fate DAPs. DAF, days after flowering.

**Supplementary Figure 1**

**A**


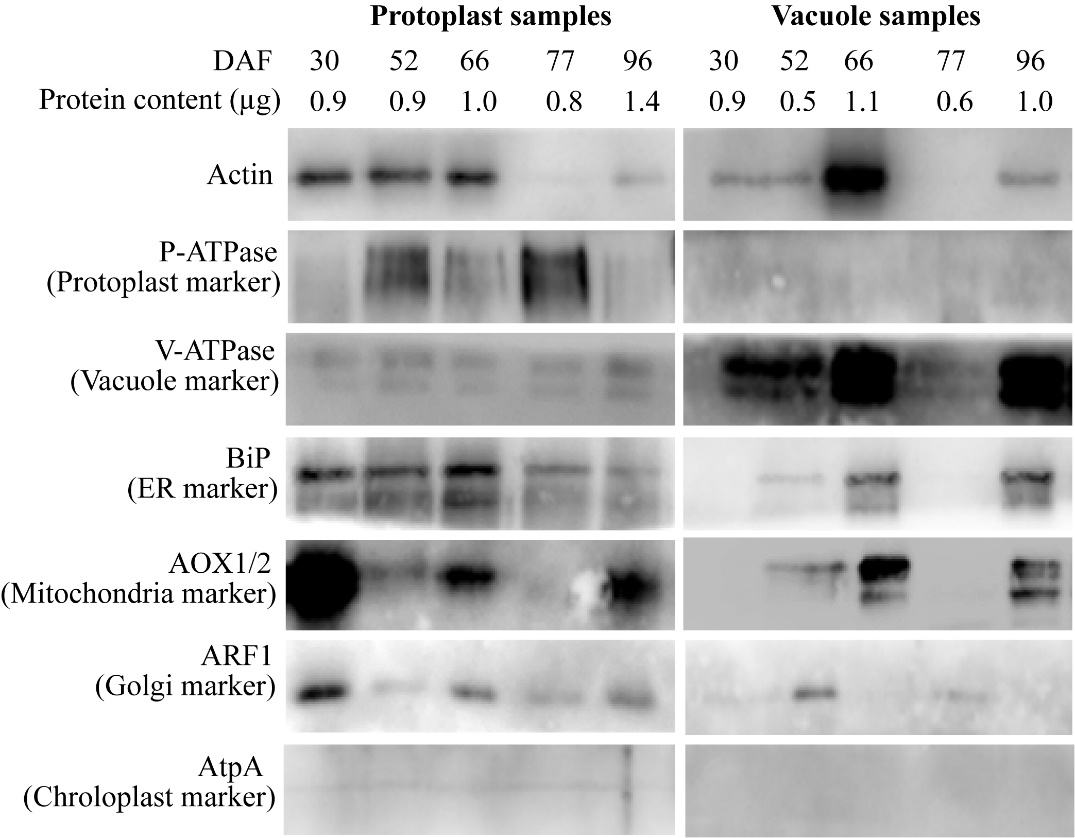


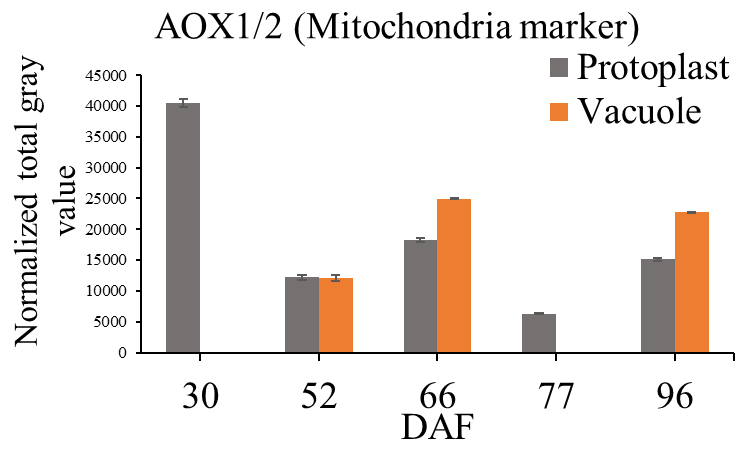

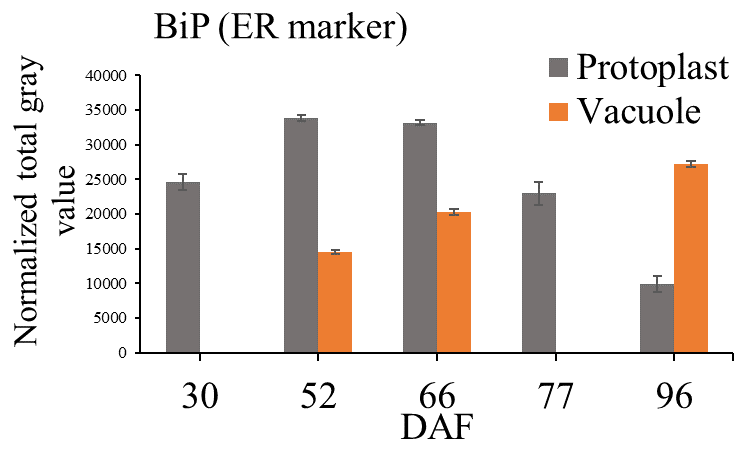


**B**


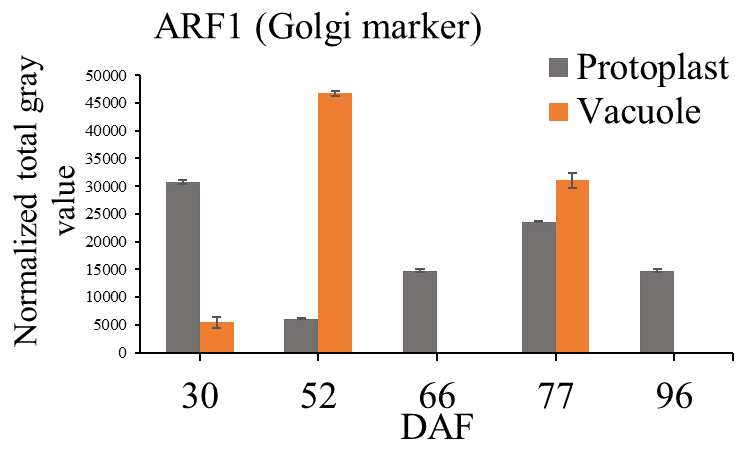

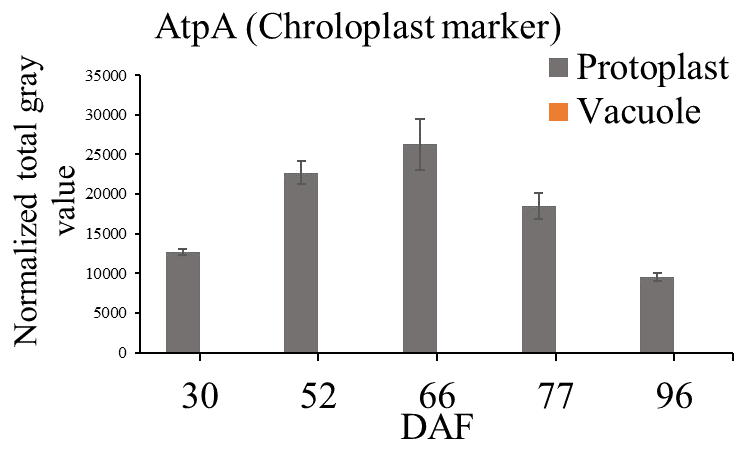


**Supplementary Figure 2**


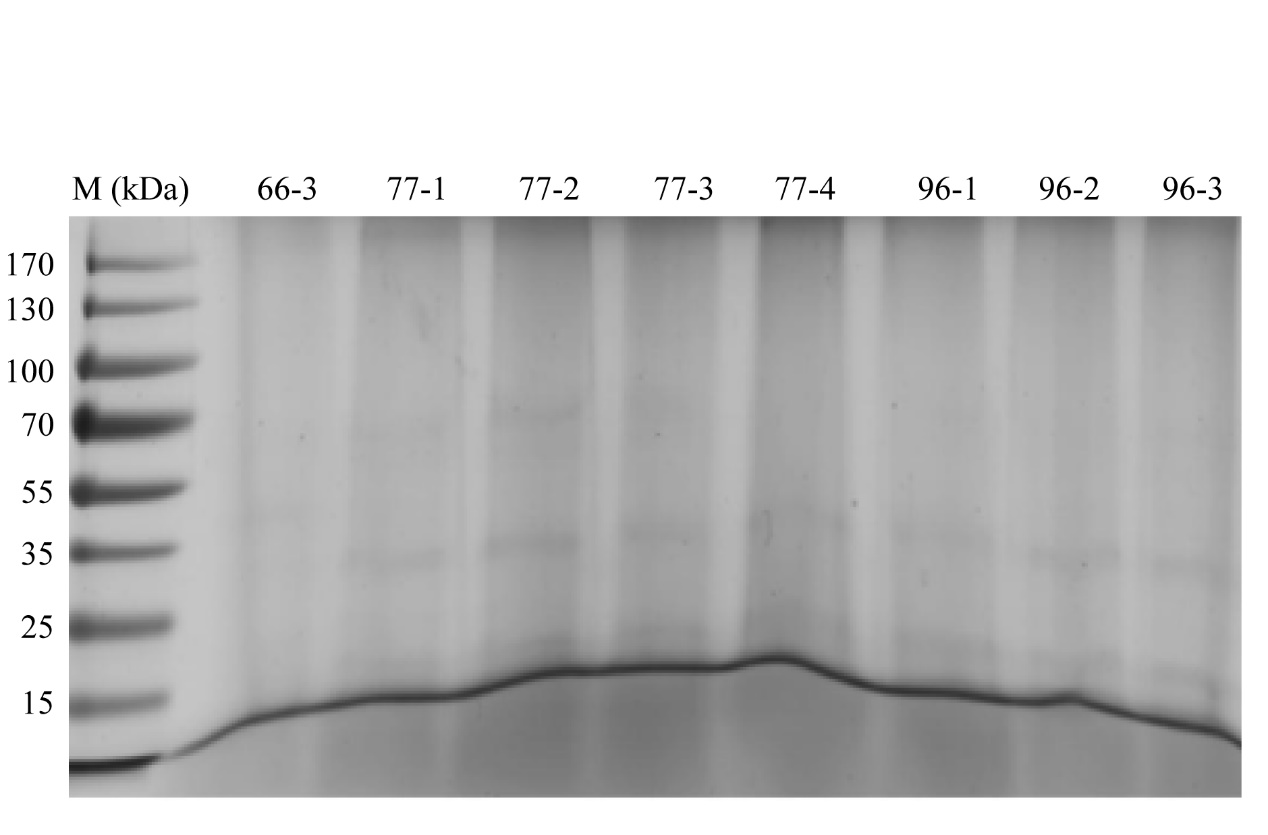


**Supplementary Figure 3**


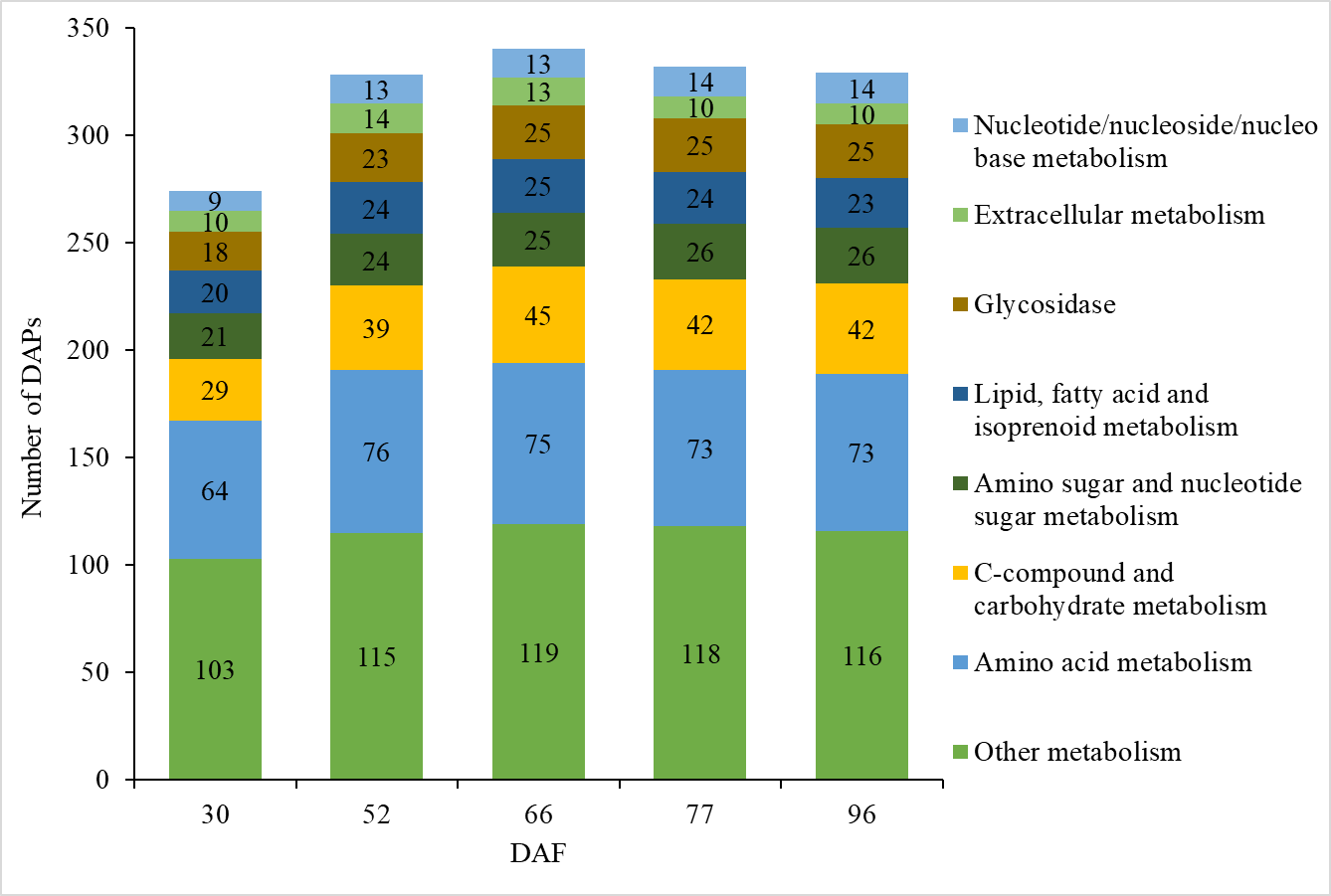

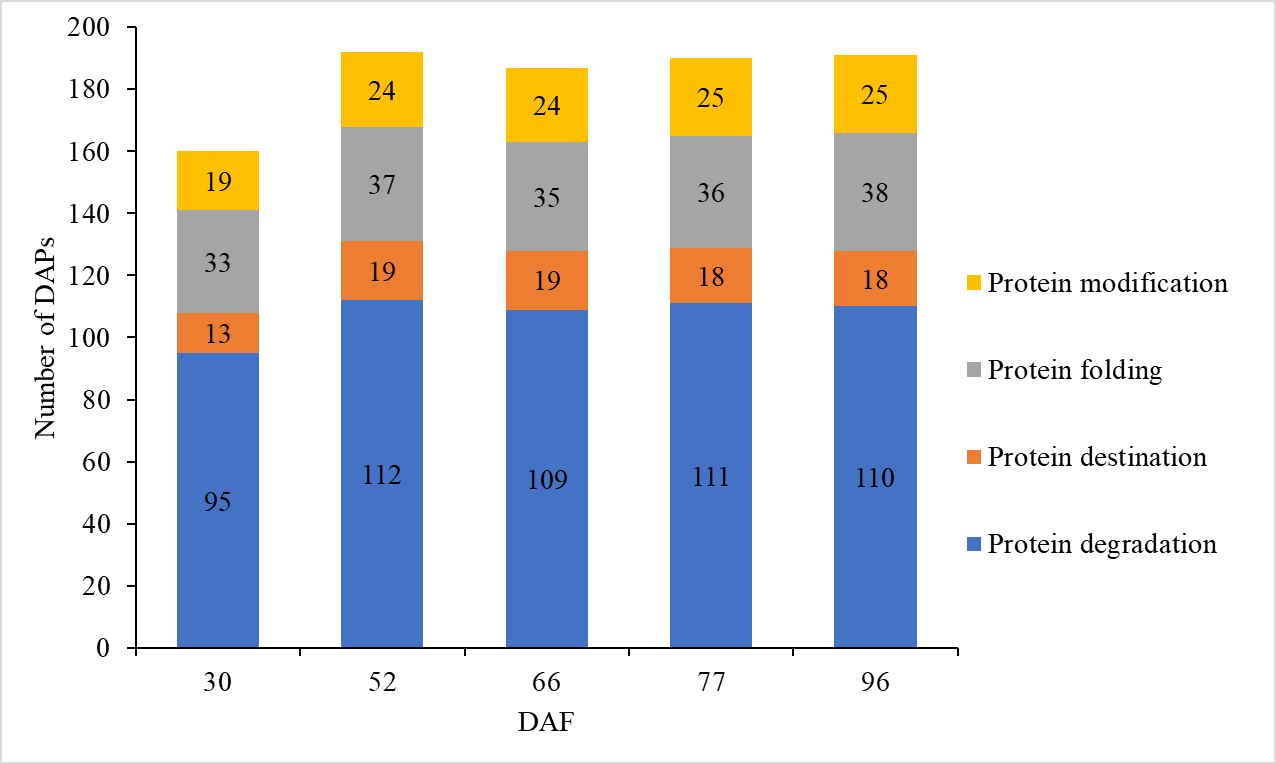


**B**
